# Green Tea Epigallocatechin-3-Gallate Suppresses Autoimmune Arthritis Through Indoleamine-2,3-Dioxygenase Expressing Dendritic Cells and the Nuclear Factor, Erythroid 2-Like 2 Antioxidant Pathway

**DOI:** 10.1186/s12950-015-0097-9

**Published:** 2015-09-15

**Authors:** So-Youn Min, Mei Yan, Sang Bum Kim, Sneha Ravikumar, Seong-Ryuel Kwon, Kamala Vanarsa, Ho-Youn Kim, Laurie S. Davis, Chandra Mohan

**Affiliations:** Division of Rheumatology, Department of Internal Medicine, University of Texas Southwestern Medical Center, 5323 Harry Hines Blvd, Bldg Y, Flr 8, Room 206 (Y8.206), Dallas, TX 75390-8884 USA; Department of Cell Biology, University of Texas Southwestern Medical Center, Dallas, TX 75390 USA; Department of Internal Medicine, Rheumatism Center, Inha University School of Medicine, Incheon, South Korea; Present address: Department of Biomedical Engineering, University of Houston, 3605 Cullen Blvd, Room 2027, Houston, TX 77204-5060 USA

**Keywords:** Green Tea (−)-epigallocatechin-3-gallate (EGCG), Collagen-induced arthritis, Regulatory T cells, Nrf-2 signaling pathway

## Abstract

**Background:**

The activity of one of the major catechins in Green Tea, the polyphenol (−)-epigallocatechin-3-gallate (EGCG), has been shown to have a variety of health benefits. Recent studies suggest that EGCG can modulate both the innate and adaptive arms of the immune system. The goal of the current studies was to examine the immunomodulatory effects and mechanisms of action of EGCG on experimental arthritis in mice.

**Methods:**

EGCG (10 mg/kg) was administered by oral gavage after CIA induction, while control mice were administered phosphate buffered saline (PBS). Disease mechanisms were studied in both groups of mice. Phenotypes were examined using repeated measure analysis of variance (ANOVA) and data from *in vitro* and *ex vivo* experiments were analyzed for significance using the Mann-Whitney *U* test.

**Results:**

EGCG treatment ameliorated clinical symptoms and reduced histological scores in arthritic mice. Serum type-II collagen-specific immunoglobulin (Ig) IgG2a antibodies were significantly lower in EGCG-fed mice compared to PBS-treated mice. EGCG significantly suppressed T cell proliferation and relative frequencies of CD4 T cells, CD8 T cells and B cell subsets including marginal zone B cells, T1 and T2 transitional B cells, while increasing the frequency of CD4^+^ Foxp3^+^ regulatory T cells (Tregs) and indoleamine‐2,3‐dioxygenase (IDO) expression by CD11b^+^ dendritic cells (DC). Splenic CD11b^+^ DC from EGCG fed mice induced an increased frequency of Tregs via an IDO-dependent mechanism in *in vitro* cultures*.* Importantly, joint homogenates from EGCG-fed mice exhibited significantly increased levels of Nuclear Factor, Erythroid 2-Like 2 (Nrf-2) and Heme oxygenase-1 (HO-1) compared with PBS-fed mice.

**Conclusions:**

This is the first report of upregulation of the Nrf-2 antioxidant pathway in EGCG-mediated immunoregulation. EGCG ameliorated experimental arthritis in mice by eliciting IDO-producing DCs, increasing frequencies of T regs and inducing the activation of the Nrf-2 antioxidant pathway. It remains to be established whether EGCG is useful for the prevention and treatment of rheumatoid arthritis and other inflammatory disorders.

## Introduction

Rheumatoid arthritis (RA) is a chronic autoimmune disease characterized by leukocyte infiltration and inflammation in the synovial membranes of joints. Inflammation contributes to pannus formation and ultimately destruction of articular bone and cartilage [1–3]. The features of disease can be severely debilitating with pulmonary, renal and cardiovascular involvement in addition to joint destruction leading to significant functional disability and increased morbidity [2].

Conventional therapies such as non-steroidal anti-inflammatory drugs (NSAIDs) and disease modifying anti-rheumatic drugs (DMARDs) along with biologics and other experimental treatments have been used to slow the clinical progression of RA [4–7]. Unfortunately, these agents have limited efficacy and serious side effects [8–11]. Thus, an important goal of RA therapy is to move toward therapies that have the potential to induce remission of disease activity and thereby control the accumulation of irreversible joint damage with fewer side effects [12, 13]. Over the last decade, the use of biologics including IL-1, IL-6 and TNF-α blockers have shown efficacy for the treatment of disease in some patients; however, their effectiveness is incomplete and there is a sizeable population of non-responders [12, 13]. Due to the limitations of conventional drugs and newer biologics, there is a growing interest in the use of herbal products with complementary activities to halt the progression of arthritis and inflammatory autoimmune diseases.

Green tea, which is a rich source of the immunomodulatory polyphenol (−)-Epigallocatechin-3-gallate (EGCG), is one of the most commonly consumed beverages in the world. EGCG has been shown to have antioxidant and anti-inflammatory effects in various animal models of autoimmune diseases [14–19]. In the past decade, the cartilage-preserving and chondroprotective action of EGCG has been verified in several studies [15, 16]. Moreover, EGCG pretreatment in an experimental immune-mediated glomerulonephritis model reduced oxidative stress and normalized levels of glutathione peroxidase and peroxisome proliferator-activated receptor-γ [17]. EGCG inhibited Th1 and Th17 cells and increased T regulatory cell (Treg) development in experimental autoimmune encephalomyelitis (EAE) models [18]. The disease-modifying effects of green tea extract on arthritis in a collagen-induced arthritis (CIA) murine model was associated with reduced inflammatory mediators including COX-2, IFN-γ and TNF-α in arthritic joints [15, 20]. Kim and colleagues have reported that Lewis rats fed with green tea extract in drinking water exhibited significantly reduced severity of adjuvant-induced arthritis (AA) and decreased serum levels of antibodies [19]. Using *in vitro* cultured primary human osteoblasts and an *in vivo* rat CIA model, another study demonstrated that EGCG was able to ameliorate arthritis in rats, associated with reduced MCP-1/CCL2 and GRO/CXCL1 synthesized by osteoblasts [21]. Although EGCG suppresses arthritis in animal models, the underlying mechanisms regulating immune cell activity have yet to be delineated. In this study, we examine the effects of EGCG on clinical arthritis, as well as the related immune mechanisms by which EGCG might exert its effects.

## Materials and methods

### Animals

Approximately 8-week-old male DBA/1 J mice (The Jackson Laboratory, Maine, USA) were maintained in groups of two to four animals in polycarbonate cages in a specific pathogen-free environment and were fed standard chow (Ralston Purina, St Louis, MO, USA) and water *ad libitum*. All experimental procedures were examined and approved by the Institutional Care and Use Committee at the University of Texas Southwestern Medical Center at Dallas.

### Oral feeding of EGCG in DBA/1 mice

DBA/1 mice were orally fed with either phosphate buffered saline (PBS) control or EGCG (10 mg/kg, Sigma, in PBS) using an oral gavage Zonde needle (Thermo Fisher Scientific, Vantaa, Finland), nine times over three weeks, beginning a week after booster immunization.

### Induction and evaluation of arthritis

Bovine type II collagen (CII) was dissolved in 0.05 N acetic acid and was emulsified (1:1 ratio) with an equal volume of complete Freund's adjuvant (CFA); both CII and CFA from Chondrex, Redmond, WA [22, 23]. Mice were immunized by tail base injection on day 0 with an emulsion of CII (100 μg) and CFA (1 mg/ml), followed by a booster injection at a separate site at the base of the tail with *an* emulsion of CII (100 μg) in incomplete freund's adjuvant (1:1) on day 14 [23]. Starting 18 days after the primary immunization, three independent observers examined the severity of arthritis three times a week for up to 6 weeks. The severity of arthritis was recorded as the mean arthritic index on a 0 to 4 scale according to the following criteria: 0 = no edema or swelling; 1 = slight edema and erythema limited to the foot or ankle; 2 = slight edema and erythema from the ankle to the tarsal bone; 3 = moderate edema and erythema from the ankle to the tarsal bone; and 4 = edema and erythema from the ankle to the entire leg [22]. The final score was an average value of three independent joint evaluations.

### Measurement of autoantibodies

Blood was collected from the orbital sinus of EGCG-treated and control mice at the peak of clinical disease. Serum specimens were stored at −20 °C until use, and anti-CII IgG1 and anti-CII IgG2a Ab levels were measured by an enzyme-linked immunosorbent assay (ELISA). Microplates were coated with 4 μg/ml of CII overnight and blocked with 1 % bovine serum albumin (BSA) from Sigma-Aldrich, St. Louis, MO and then incubated with sera at a dilution of 1:16,000. Bound total or CII-specific IgG1 or IgG2a were detected by incubation with horseradish peroxidase (HRP)-conjugated goat anti-mouse IgG1 or IgG2a-specific antibodies (cat # A90-205P and A90-207P from Bethyl Laboratories, Inc., Montgomery, TX) for 1 h. Then the plates were washed with phosphate-buffered saline with Tween 20 buffer (PBST) and developed with 3,3',5,5'-tetramethylbenzidine (TMB) substrate according to the manufacturer’s instructions (Sigma-Aldrich). The reaction was terminated with 4.5 N sulfuric acid (H_2_SO_4_). The optical density (OD) values were measured at 450 nm using an Automatic Microplate Reader (BLx808, BIO-TEK, Winooski, Vermont).

### Flow cytometry and antibodies

Red blood cells were depleted from splenocytes and lymph node cells using lysis buffer which contained 10 mM potassium bicarbonate (KHCO_3_), 0.15 M ammonium chloride (NH_4_Cl) and 0.1 M ethylenediaminetetraacetic acid (EDTA), pH 7.2, and single cell suspensions were prepared and flow cytometric analysis was performed using a FACSCalibur (BD Biosciences, San Jose, CA) with BD CellQuest Pro Software (BD Biosciences) and the data was analyzed using FloJo Software (FlowJo, LLC, Ashland, OR). For analysis of lymphocytes the following rat anti-mouse antibodies were used: CD4-PerCP-Cy5.5 (clone RM4-5), CD8-PE (clone 53–6.7), CD21/35-FITC (clone 7G6) and CD23-Biotin (clone B3B4) with Streptavidin- allophycocyanin (APC); all antibodies and second step reagents from BD Biosciences. Tregs were identified using anti-mouse FoxP3-FITC (clone FJK-16a; eBioscience) and CD25-APC (clone 3C7; Biolegend). CD11b^+^ cells were identified using rat anti-mouse CD11b-PerCP (clone M1/70; BD Biosciences) and indoleamine‐2,3‐dioxygenase (IDO)-positive cells were detected with rat anti-mouse IDO (clone mIDO-48; Biolegend) followed by FITC goat anti-Rat Ig secondary antibody (cat# 554016, BD Biosciences).

### Intracellular Staining for IFN-γ and TNF-α

Single cell suspensions isolated from draining lymph nodes were stimulated with 25 ng/ml PMA (Sigma) and 250 ng/mL ionomycin (Sigma). GolgiStop (BD Biosciences) was added and the cells were harvested after 5 h of culture. Cells were first stained extracellularly with anti-CD4 PerCP-Cy5.5-conjugated (clone RM4-5) antibody, then fixed and permeabilized with Perm/Fix solution, and finally stained intracellularly with anti- IFN-γ FITC-conjugated (clone XMG1.2), and anti- TNF-α PE-conjugated (clone MP6-XT22) antibodies. Directly conjugated isotype-matched rat anti-mouse antibodies were used as controls for nonspecific staining. All reagents for fixation and staining were from BD Biosciences and the protocol was carried out following the manufacturer’s instructions.

### ^3^H-thymidine incorporation in mouse spleen and draining lymph nodes cells

Draining inguinal lymph nodes (dLNs) were aseptically excised, minced and single cell suspensions were cultured in triplicate in RPMI 1640 containing fetal calf serum (10 % vol/vol), 2-mercaptoethanol (20 μM), L-glutamine (1 % wt/vol), penicillin (100 U/mL), and streptomycin (100 μg/mL) in the presence or absence of CII (Chondrex) or rat anti-mouse CD3 antibody (clone 17A2; BD Biosciences). During the last 16–18 h of the three-day assay, cells were pulsed with 1 uCi of [^3^H]-thymidine (Perkin Elmer, Waltham, MA) per well. The incorporation of [^3^H]-thymidine was determined using a Betaplate scintillation counter (Perkin-Elmer, Waltham, MA).

### Preparation of tissue homogenates and western blotting

Hind paws and knees were removed from the sacrificed mice, frozen in liquid nitrogen and lysed using a buffer containing 20 mM Tris–HCl (pH 7.5), 150 mM NaCl, 1 mM Na2EDTA, 1 % Triton X-100, protease inhibitor cocktail (Complete Mini, Roche, Indianapolis, IN) and phosphatase inhibitor cocktail (PhosSTOP, Roche, Indianapolis, IN). The crude extract was then sonicated for 30 s. The homogenate was centrifuged at 20,000 X g for 15 min, and the resulting supernatant was collected. The total protein content of samples was quantified using the Bradford assay (Sigma). 10 μg of each sample was resolved by sodium dodecyl sulfate polyacrylamide gel electrophoresis (SDS-PAGE) using a 4–15 % Mini-PROTEAN TGX Precast Gel (Biorad, Hercules, CA), transferred to a polyvinylidene difluoride (PVDF) membrane (Biorad, Hercules, CA), and probed with primary antibodies including rabbit antibody to Nuclear Factor, Erythroid 2-Like 2 (anti-Nrf2; catalog no. ab31163), rabbit antibody to phosphorylated Nrf-2, anti-p-Nrf2 (ab76026) and a mouse IgG1 monoclonal antibody that recognizes mouse heme oxygenase-1, anti-HO-1 (ab13248, clone HO-1-1); all from Abcam and all have been previously shown to react with murine proteins. The loading control was an anti-GAPDH (clone 6C5; Advanced ImmunoChemical) which reacts to mouse and other species. Appropriate HRP-conjugated secondary antibodies which included goat anti-rabbit or goat anti-mouse (Jackson ImmunoResearch) were used at 1:5000 and detected with the SuperSignalWest Femto Chemiluminescent Substrate Kit (Thermo Scientific). Protein expression levels were visualized and quantitated using the gel documentation system, G:BOX (Syngene, Frederick, MD).

### Measurement of IDO Enzymatic Activity

The indoleamine‐2,3‐dioxygenase (IDO) enzyme activity assay was performed as previously reported [24] with some modifications. In brief, freshly isolated DCs were washed, resuspended in sterile Hank’s Balanced Salt Solution (HBSS; Sigma-Aldrich) containing 500 uM tryptophan (Sigma-Aldrich), and incubated for 4 h. The supernatants were then harvested and assayed for kynurenine. For the assay, 30 ul of 30 % trichloroacetic acid was added to 60 ul of culture supernatant and the mixture was vortexed and centrifuged at 10,000 X g (12,000 rpm) for 5 min. Then, 40 ul of supernatant was added to an equal volume of Ehrlich reagent (5 ml of glacial acetic acid with 100 mg *P*-dimethylamino-benzaldehyde). The OD was measured at 492 nm using a NanoDrop (LMS). Purified _L_-kynurenine (0–500 uM; Sigma-Aldrich) was used as the standard.

### Fluorescence microscopy

Spleens from mice were collected, embedded in Tissue-Tek Optimal Cutting Temperature (O.C.T.) compound and snap-frozen in liquid nitrogen. Cryosections (6 μm thick) were fixed with 4 % paraformaldehyde, blocked with 10 % horse serum for 30 min and stained with various antibodies. Anti-Foxp3-FITC (clone FJK-16a; e-Bioscience) and anti-IDO (clone mIDO-48; Biolegend) or Rat IgG2b isotype control (Biolegend) were detected with goat anti-rat Alexa-555 secondary (catalog # A-21434; Invitrogen) and anti-APC-CD11b (clone M1/70; BD Bioscience). Fluorescence images were acquired using an LSN510 confocal microscope (Carl Zeiss, Oberkochen, Germany). For quantification of immunofluorescence (IF) 10 representative high-powered fields per slide were assessed from at least 4 to 5 samples/group.

### Detection of Cytokine Production by Enzyme-Linked Immunosorbent Assay (ELISA)

Serum specimens were assayed for IL-1β, IL-6, IFN-γ, TNF-α and IL-10 by ELISA using Duoset assay kits from R&D Systems and following the manufacturer’s instructions.

### Co-culture of DC with T cells

Mice were sacrificed seven weeks after immunization. The spleens obtained from the mice were treated with RPMI 1640 (Invitrogen) containing dithiothreitol (DTT) and EDTA for 90 min at 37 °C to remove the epithelial cells and then washed with HBSS and digested with DNase. CD11b^+^ DCs cells were separated from splenocyte suspensions from EGCG-fed or PBS-fed mice using a mouse CD11b positive selection kit (catalog # 18770; Stem Cell, Canada) and were co-cultured with CD4^+^CD25^−^ T cells isolated from EGCG-fed mice for 3 days in the presence or absence of CII (10 μg/ml, Chondrex Inc, USA). Before the stimulation with CII, the cells were pretreated with the IDO-specific inhibitor 1-methyl dL tryptophan (1-MT), obtained from Sigma-Aldrich, for two hours. To measure the amount of intracellular Foxp3 in CD4^+^CD25^+^ T cells, cells were stained using a Regulatory T Cell Staining Kit (eBioscience, San Diego, CA, USA) as described in flow cytometry above.

### Histology of joint tissues

Hind paws and knees were obtained from each mouse, the skin was trimmed, and the joints were fixed in 10 % phosphate-buffered formalin for 1 day, decalcified in 15 % EDTA for 3 weeks and embedded in paraffin. Tissue sections (6 μm) were prepared and stained with hematoxylin, eosin and safranin O. Inflammation and joint damage were assessed by scoring five parameters. Disease was scored on a scale of 0 to 3 for inflammation ranging from no inflammation to severe inflammation, loss of proteoglycans ranging from fully stained to destained cartilage, cartilage destruction ranging from appearance of dead chondrocytes to complete loss of the articular cartilage, and was scored on a 0 to 5 scale for loss of bone ranging from no damage to complete loss of bone structure. For histology of both knee and paw, a composite score was calculated by summing the individual parameters.

### Inhibition of IDO with 1-Methyl-dL-Tryptophan

1-MT was obtained from Sigma-Aldrich and was prepared as a 20-mmol/l stock in 0.1 N NaOH (pH 7.4) and stored at −20 °C in the dark. EGCG fed mice were given 2 mg/ml 1-MT solution, supplemented with aspartame using foil-wrapped, standard autoclaved drinking bottles. Mice drank an average of 5 ml/d, and water was replaced as needed for 3 weeks.

### Statistical analysis

Phenotypes were examined using repeated measures one-way analysis of variance (ANOVA), with treatment and time as fixed factors and mouse number as the random factor. Data from *in vitro* and *ex vivo* experiments were analyzed for statistical significance using the Mann-Whitney *U* test (GraphPad Prism 6, GraphPad Software, Inc., San Diego, CA, USA). A *p* value < 0.05 was taken as the level of significance. In all experiments, * indicates *P* < 0.05, ** *P* < 0.01, and *** *P* < 0.001.

## Results

### EGCG treatment reduces disease activity in collagen-induced arthritis

**Fig. 1:**
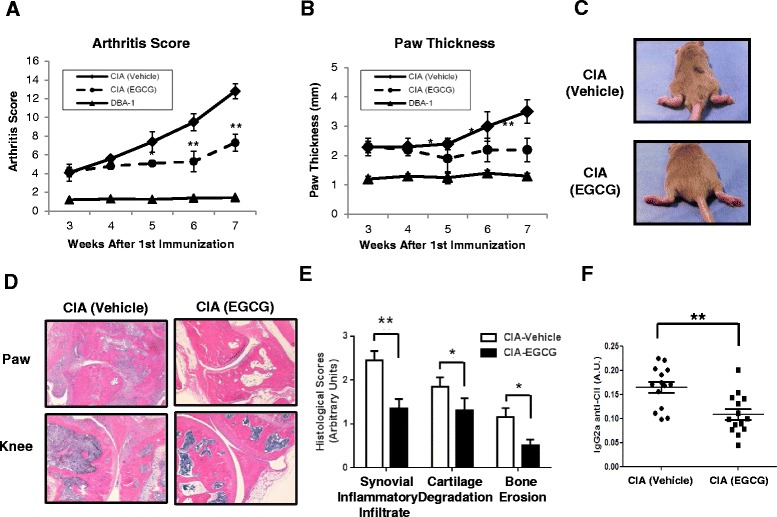
CIA induction and EGCG administration in CII-immunized DBA/1 J mice. **a** Effects of EGCG on arthritis score of CIA mice. DBA1/J mice (DBA-1) were untreated or immunized with 100ug CII in complete CFA. A booster injection of 100 ug of CII was given on day 14 to induce CIA in mice. Beginning on day 21 post CII immunization, 10 mg/kg EGCG was administered orally using oral gavage for nine successive times every two days over the course of three weeks. PBS was given to the CIA control group over the same time period. Mice were sacrificed on day 49 for immunophenotypic analysis and histopathological examination, *P < 0.05, **P < 0.01. *N* = 4 to 5 mice per group for each experiment, with a total of 14 mice per group; *P < 0.05; **P < 0.01. *N* = 14 mice for each group. Data shown is representative of three independent experiments. **b** Effects of EGCG on paw swelling of CIA mice. Mice were untreated DBA-1 or immunized with CII as described above and treated with PBS (vehicle) or EGCG. All data are shown as mean ± SEM of three independent experiments. *N* = 4 to 5 mice per group for each experiment, with a total of 14 mice per group. **c** Representative photographs depicting vehicle-fed (upper) and EGCG-fed CIA mice (lower) on day 49 after 1^st^ immunization. **d** Histopathology of paw and knee joints from representative CIA mice treated with vehicle (left) or EGCG (right). Joints were harvested on day 49 and joints were decalcified and stained with H&E (original magnification × 40). Five mice were examined per group. **e** Values of histological scores are shown as mean ± SEM derived from five mice per group for synovial inflammatory infiltrate, cartilage degradation and bone erosion. **f** Serum titers of CII-specific IgG2a were detected by ELISA in the individual EGCG (*n* = 14) or vehicle-fed mice (*n* = 14). Serum samples were obtained on day 49 post first CII immunization. The results were calculated in arbitrary units and are expressed as mean ± SD of three independent experiments, ∗∗*P* < 0.01

We determined whether EGCG modulated disease activity in a murine collagen-induced arthritis (CIA) model. DBA/1 mice were immunized on days 0 and 14 with bovine type II collagen (CII), as detailed in the methods. Mice were fed with EGCG nine times over three weeks with 10 mg/kg, starting on day 21 after induction of arthritis throughout the disease course and vehicle-fed or EGCG-fed mice were observed for 49 days for the development of clinical arthritis (Fig. 1). Mice treated with PBS (vehicle control) developed the typical signs of CIA both in terms of clinical score and paw swelling seven weeks after the initial immunization (Fig. 1a, b). In contrast, EGCG-treated mice displayed a significant decrease in severity of arthritis and paw thickness compared to animals treated with vehicle alone (Fig. 1a, b). As expected, vehicle-fed CIA mice developed severe symptoms of arthritis including marked swelling, redness, and erythema of the hind paws and the forepaws. In contrast, EGCG-fed mice exhibited markedly reduced clinical manifestations of fully developed CIA (Fig. 1c).

The observed clinical paw inflammation was also confirmed by assessment of H&E stained histological sections of mouse hind paws. The histological images of the paws shown in Fig. 1d are representative of mice in each group at day 49 when disease was found to be at its maximum. Joint tissue samples from arthritic vehicle-fed mice revealed the expected histopathological changes, including marked synovial hyperplasia, erosion, and loss of articular cartilage and bone. In this group, there was extensive cartilage and bone erosions with massive infiltration of polymorphonuclear and mononuclear leukocytes as indicated by the elevated histopathological score (Fig. 1e). The joint swelling and pannus formation appeared to be dependent on disease severity. A similar analysis of arthritic joints from the EGCG-fed group showed a marked reduction in the number of infiltrating leukocytes, with less visible cartilage or bone erosion when compared with the vehicle-fed CIA mice (Fig. 1d, e). Thus, these results indicate that EGCG reduced infiltration of inflammatory cells and diminished the severity of arthritis as assessed by clinical and histological scores. Consistent with previous reports, our results demonstrate that the oral administration of EGCG successfully ameliorates disease activity in an inflammatory arthritis model.

### EGCG reduces serum levels of type II-collagen-specific IgG2a antibodies

Previous studies have shown that humoral immunity plays an essential role in the pathogenesis of CIA [25, 26]. Our results indicate that, in EGCG-treated mice CII-specific IgG2a antibodies were dramatically reduced (*P* < 0.01) when compared to vehicle-fed CIA mice (Fig. 1f). In contrast, the titers of anti-CII specific IgG1 antibodies were increased in EGCG-treated mice compared with vehicle-treated mice (*P* < 0.5; data not shown). These results suggest an important role for EGCG in modulating B cell responses, as well as Th1/Th2 balance in the ongoing immune response.

### EGCG treatment reduces serum and joint inflammatory cytokine production

**Fig. 2:**
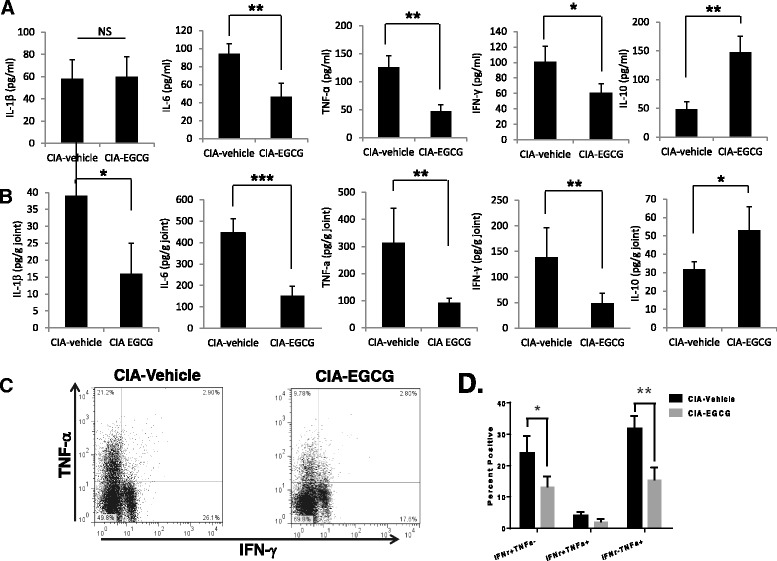
Proinflammatory cytokine expression in EGCG-fed CIA mice. Changes in the levels of IL-1β, IL-6, TNF-α, IFN-γ and IL-10 were measured in **a** sera and **b** joint homogenates using ELISA assays and joint samples were normalized by protein concentration. All data are shown as mean ± SEM from three independent experiments involving 4 to 5 mice per group (*P < 0.05; **P < 0.01; ***P < 0.001). **c**,**d** The draining lymph node cells from CIA mice treated with EGCG or vehicle were cultured with PMA (25 ng/ml) and ionomycin (250 ng/ml) in the presence of a protein export inhibitor for 5 h. Frequencies of TNF-α and IFN-γ expressing CD4^+^ T cells were assessed by intracellular staining and flow cytometric analysis. Frequencies of TNF-α and IFN-γ were significantly reduced in the EGCG-fed mice as compared to controls whereas frequencies of TNF-α^+^ IFN-γ^+^ cells were similar between the two groups. The results shown were from one representative experiment of three independent experiments

Previous reports have shown that arthritis is associated with the presence of Th1 CD4^+^ T effector cells secreting high levels of IFN-γ while there is an absence of Th2 effectors in the arthritic synovium in murine CIA models and RA [20, 25, 27]. IFN-γ induces activation of macrophages that produce proinflammatory cytokines, such as TNF-α and IL-1β which are abundant in arthritic joints in animal models and RA [25, 28, 29]. We therefore measured pro-inflammatory cytokines in the serum and knee homogenates of the experimental mice (Fig. 2). At day 49 after induction of CIA, EGCG significantly reduced serum levels of IL-6, TNF-α, and IFN-γ compared to vehicle-fed control CIA mice. However, there was no statistical difference in the production of IL-1β while IL-10 levels were elevated in the serum of EGCG-fed mice (Fig. 2a). Similar results were obtained when pro-inflammatory cytokines were measured in knee homogenates (Fig. 2b). The levels of IL-1β, IL-6, TNF-α and IFN-γ were significantly lower and IL-10 was higher in EGCG-fed mice when compared with vehicle-fed CIA mice. We also quantitated the frequency of cells producing Th1 cytokines including IFN-γ and TNF-α in the draining lymph nodes of EGCG-fed mice and vehicle-fed control mice (Fig. 2c, d). In concordance with the results for the serum and joint cytokine profiles, Th1 cell frequencies were significantly reduced in the EGCG-fed mice as compared to the controls. Our results suggest that consumption of EGCG limited inflammatory Th1 cell numbers and cytokines, which may have contributed to the altered anti-CII antibody isotype profiles and reduced disease severity.

### Alteration of CIA-induced immune cell populations by dietary EGCG

**Fig. 3:**
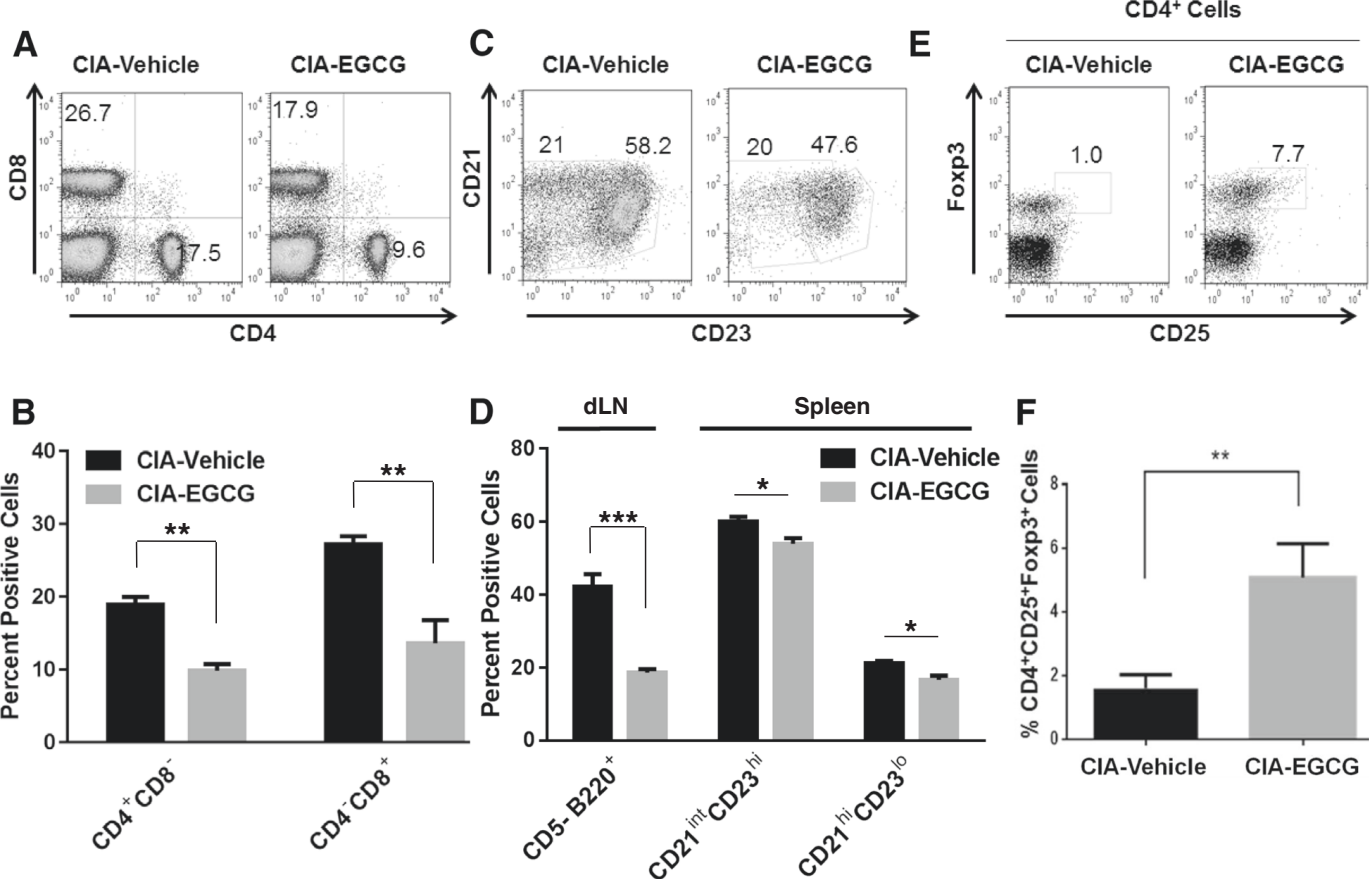
EGCG suppresses lymphocyte responses to antigen stimulation in CIA. Subsets of T cells from draining lymph nodes (dLN) and B cells from spleen in vehicle-fed or EGCG-fed CIA mice were examined for frequencies by flow cytometry in three independent experiments with 4 to 5 mice per group. **a**,**b** Representative data for major T lymphocyte subsets of CD4^+^ (CD4 ^+^ CD8 ^-^) and CD8^+^ (CD4^−^ CD8 ^+^) T cells from the dLN are shown. **c**,**d** dLN B cells (CD5^−^B220 ^+^) and splenic B cell subsets were assessed including splenic marginal zone B cells (B220 ^+^ CD23^−^CD21 ^+^) and splenic follicular B cells (B220 ^+^ CD23 ^+^ CD21^−^). The absolute number of marginal zone B cells (5.0 × 10^7^ cells ± 0.8 vehicle-fed vs 1.5 × 10^7^ cells ± 0.1 EGCG-fed, *P* < 0.01) and follicular B cells (1.9 × 10^7^ cells ± 0.2 vehicle-fed vs 0.3 × 10^7^ cells ± 0.04 EGCG-fed, *P* < 0.01) in CIA mice were significantly reduced in numbers following EGCG treatment. **e**,**f** Representative data shows EGCG treatment increased the number of CD4^+^Foxp3^+^ T cells recovered from the dLN as compared to vehicle-fed CIA mice

RA is characterized by chronic inflammation of the synovium which could result in part from the infiltration of activated immune cells including CD4^+^ T cells, B cells, and antigen presenting cells including DC and macrophages [2, 20, 27, 29]. To identify the changes in immune cell populations after EGCG supplementation, we conducted flow cytometric analysis (Fig. 3). EGCG reduced the frequencies of B cells (CD5^−^B220^+^) and the major T-cell subsets (CD4^+^CD8^−^ and CD4^−^CD8^+^) in the dLNs on day 49 in CIA (Fig. 3a, b, d). We next examined the frequencies of different splenic B cell subsets including the follicular (Fo, CD21^int^CD23^hi^) and marginal zone (MZ, CD21^hi^CD23^lo^) B cells [30]. EGCG treatment significantly reduced the frequencies of Fo and MZ B cells in the spleen (Fig. 3c, d). Finally, mice treated with EGCG had significantly increased frequencies of CD4^+^CD25^+^Foxp3^+^ T-regulatory (Treg) cells in the dLNs (Fig. 3e, f). There was an average 2-fold increase in Treg frequencies in EGCG-treated mice compared to the vehicle-fed CIA mice.

### EGCG treatment attenuates T cell proliferation in CIA

**Fig. 4:**
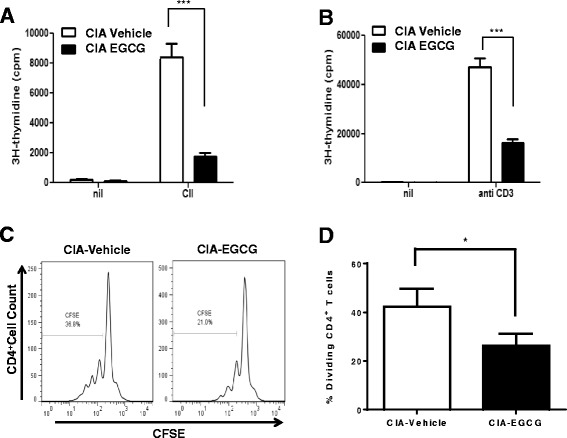
EGCG treatment attenuates T cell proliferation in CIA. Cells from the dLN of CIA mice sacrificed seven weeks after CIA induction were cultured for 72 h in the absence or presence of **a** 10 ug/ml CII or **b** anti-CD3 antibody. After pulsing with 1 uCi of [^3^H] thymidine per well for the last 18 h, proliferation was measured as [^3^H] thymidine incorporation in counts per minute (cpm). Results shown are representative of data from 4 to 5 mice per group from three independent experiments with similar numbers of mice; ∗∗∗*P* < 0.001. **c**,**d** 5,6-carboxyfluorescein succinimidyl ester (CFSE)-labeled dLN cells were cultured with type II collagen for 72 h, followed by flow cytometric analysis of CD4^+^ T cells; ∗*P* < 0.05. Data are representative of one sample (histograms) and one experiment (graph) from three independent experiments with 4 to 5 mice per group per experiment

Activation and cytokine production by CD4^+^ T cells play an important role in the pathogenesis of inflammatory arthritis. Upon antigen encounter, clonal expansion of Ag-specific T cells is a prerequisite for the initiation and development of the T-cell mediated immunopathological features [20, 22, 23]. We next determined whether the EGCG-induced reduction in severity of murine CIA might be mediated by its impact on Ag-specific T-cell responses. Draining lymph nodes (dLN) were collected from collagen-immunized/boosted DBA/1 mice after 49 days following vehicle or EGCG treatment. Cells cultured in the absence of CII, exhibited basal levels of DNA synthesis, as measured by incorporation of [^3^H]-thymidine, that did not differ between the vehicle-fed and EGCG-fed groups (Fig. 4a, b). In contrast, cells from vehicle-fed mice cultured in the presence of CII exhibited a robust antigen recall response, which was significantly reduced in cells derived from EGCG-fed mice (Fig. 4a). Mice treated with EGCG also demonstrated significantly reduced T cell DNA synthesis when stimulated with anti-CD3 (Fig. 4b). Consistent with the above results, CFSE assays demonstrated that EGCG reduced CD4^+^ T cell proliferation (Fig. 4c, d). These results indicate that administration of EGCG attenuates antigen-induced T cell proliferation in murine CIA.

### EGCG induction of IDO expression by CD11b^+^ dendritic cells in CIA

**Fig. 5:**
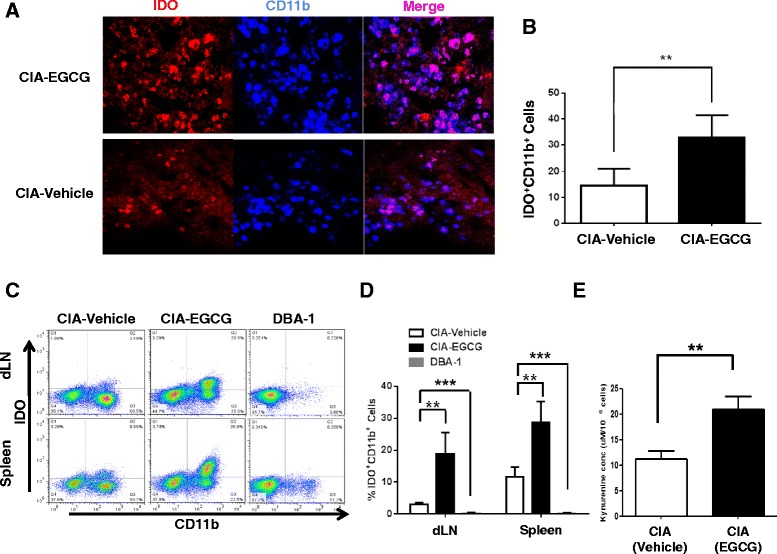
EGCG treatment increases IDO expression and activity in CD11b^+^ cells. CIA mice were fed with EGCG or PBS (vehicle) for 7 weeks after CIA induction and (**a**,**b**) dLN sections were stained for CD11b and IDO, respectively (magnification: X200). Photomicrographs and graph shown are representative of three independent experiments with 4 to 5 mice per group. (**c**,**d**) Expression of IDO in CD11b^+^ cells from dLN and spleen were analyzed by flow cytometry. Data are representative of three independent experiments with a total of 14 mice per group. (**e**) Functional IDO activity in freshly isolated CD11b^+^ cells was measured by the production of kynurenine in the culture supernatants of sorted DC as detailed in the methods. CD11b^+^ DC from spleens were sorted and incubated in HBSS for 4 h. Studies were performed in triplicate and repeated at least twice with similar results; in total seven mice were examined from each group

IDO is an intracellular enzyme that catabolizes the essential amino acid tryptophan, expressed in monocytes, dendritic cells (DC) and macrophages. IDO has been shown to suppress T cell proliferation by mechanisms that remain to be elucidated [31]. Active IDO can be induced by IFN-γ, endotoxin, a CTLA-4 fusion protein via CD80/CD86 ligation and the combination of PGE_2_ and TNF-α [24, 32]. A previous study demonstrated that IDO expressing DC in Peyer’s patches play an essential role in the induction of oral tolerance in CIA [31]. To examine whether DC in EGCG-fed mice express IDO as one mechanism for subduing CIA, we performed immunofluorescence staining for IDO and CD11b on tissue sections of dLN obtained on day 49 after disease induction. As shown in Fig. 5a, b, IDO staining (red) was clearly increased in a substantial proportion of the CD11b^+^ DC (blue) from the dLN of EGCG-fed mice as compared to the vehicle controls. Consistent with the above results, flow cytometric analysis of the dLN and spleens revealed that the CD11b^+^IDO^+^ DC were significantly increased after EGCG treatment (Fig. 5c, d). We next examined whether the DC-expressed IDO was enzymatically active. As shown in Fig. 5e, the culture supernatants of CD11b^+^ DC isolated from EGCG-fed mice contained high levels of kynurenine, the first catabolite in the tryptophan metabolic pathway, compared to the controls. These studies demonstrate that the EGCG-induced IDO expressed by CD11b^+^ DCs was functionally active.

### IDO-expressing CD11b^+^ DC are required for CD4^+^CD25^+^ Treg generation *in vitro*

**Fig. 6:**
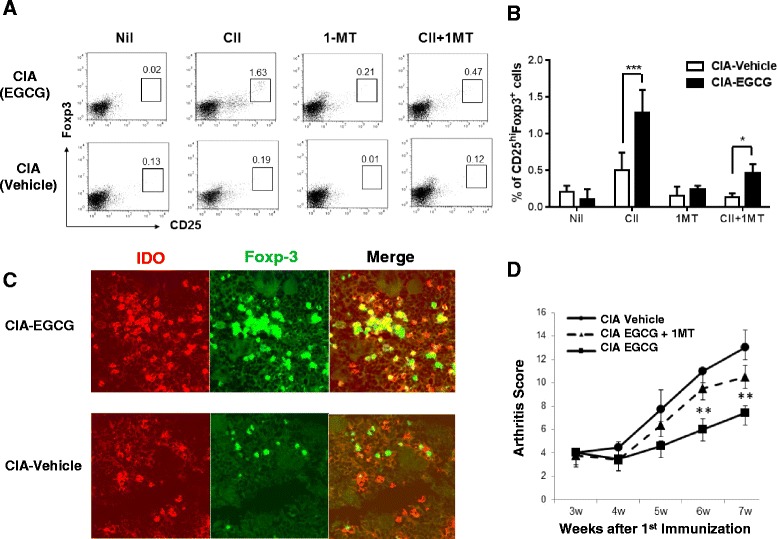
IDO^+^CD11b^+^ DC induce antigen-specific CD4^+^CD25^+^ Tregs and are juxtaposed to Tregs. Increased generation of CD4^+^CD25^+^ Foxp3^+^ T cells is mediated through an indoleamine 2,3-dioxygenase (IDO)-dependent mechanism. **a**,**b** For regulatory T cell induction, isolated CD4^+^CD25^−^ T cells (1 × 10^5^/well) were cultured for three days with CD11b^+^ DC (1 × 10^5^/well) from EGCG-fed or vehicle-fed CIA mice in the absence or presence of type II collagen (CII, 10 μg/ml). 1-MT was added to the indicated cultures. CD4^+^ cells were first gated and then plotted as CD25 versus Foxp3 as determined by flow cytometry. Numbers represent the percentage of CD4^+^ CD25^+^ Foxp3^bright^ positive cells and are representative of three independent experiments. **c** Immunofluorescent analysis of IDO and Foxp3 expression in the spleen. Spleens from EGCG-fed or vehicle-treated CIA mice were fixed and stained with antibodies specific for mouse IDO (red) and Foxp3 (green); original magnification, X200. Photomicrographs shown are representative of three independent experiments. **d** Arthritis scores for vehicle-fed, EGCG-fed, or EGCG-fed mice given 2 mg/ml of 1-MT solution in drinking water bottles for three weeks. Values are presented as the mean ± standard error of the mean, and represent two independent experiments; in total eight mice were examined for each group

We next investigated whether the CD11b^+^ DC induced by EGCG were more potent in generating regulatory T cells (Treg). Highly purified CD4^+^CD25^−^ T cells isolated from the spleen of EGCG-fed mice (purity > 97 %) were cocultured with CD11b^+^ DC from EGCG or vehicle-fed control CIA mice for 3 days in the presence or absence of CII. Figure 6a,b shows that the frequency of CD4^+^CD25^+^Foxp3^bright^ T cells was increased when CD4^+^CD25^−^ T cells were co-cultured with CD11b^+^ DC from EGCG-fed CIA mice in the presence of CII antigen than when CD4^+^CD25^−^ T cells were co-cultured with CD11b^+^ DC from vehicle-fed CIA mice. To determine whether IDO was mechanistically involved in DC-mediated antigen-specific Treg generation after EGCG treatment, 1-MT, an IDO inhibitor, was added to the CII antigen-stimulated cultures. Indeed, 1-MT abrogated the increase in the proportion of CD4^+^CD25^+^ Foxp3^+^ T cells induced by CD11b^+^ DC from EGCG-fed CIA mice (Fig. 6a, b) which correlated with arthritis scores (Fig. 6d). Thus, our findings demonstrate that highly purified CD4^+^CD25^−^ T cells can be converted into T regulatory cells (Tregs) by splenic DC obtained from EGCG-fed mice by antigen-specific stimulation with CII through an IDO-dependent mechanism. Previous studies have demonstrated the presence of IDO expressing cells frequently juxtaposed to Tregs in other disease models. For example, IDO expressing myeloid DC and macrophages in cutaneous granulomas were adjacent to Foxp3^+^ cells [33]. We next investigated whether EGCG treatment promoted a physical association between IDO expressing CD11b^+^ DC and Tregs in the spleen. Immunofluorescence analysis of EGCG-treated mice revealed that Foxp3 and IDO expressing CD11b^+^ cells were present and adjacent to each other in spleens to a greater extent than in control vehicle-treated mice (Fig. 6c). Thus, these studies correlate with the *in vitro* observation that IDO expressing CD11b^+^ DC can augment Treg numbers.

### EGCG treatment suppresses arthritis by enhancing Nrf2 activity through an IDO-dependent mechanism *in vivo*

**Fig. 7:**
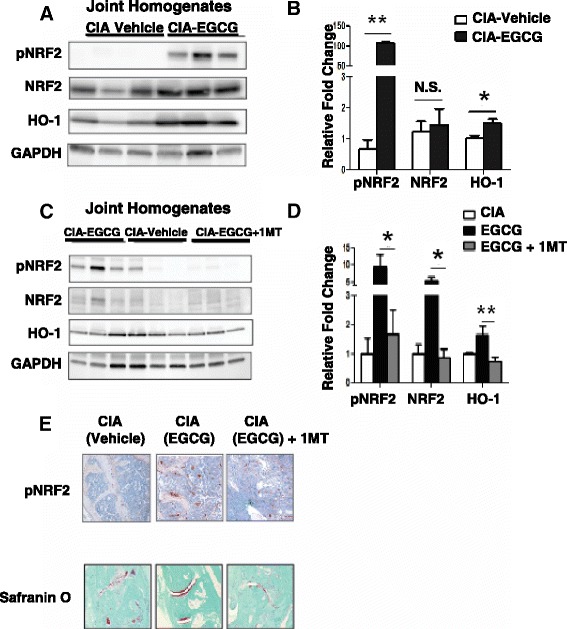
EGCG induces IDO-dependent Nrf-2 activation in joints of CIA mice. **a** Representative western blots for Nrf-2, phosphorylated Nrf-2 (pNRF2), and HO-1 expression in joint homogenates. **b** Densitometry results for pNrf2, Nrf2 and HO-1 as shown in (**a**) as relative fold change compared to the GAPDH loading control. Results are the mean ± SD of replicate samples, representative of three independent experiments. **c** Representative western blots for Nrf-2, pNRF2, and HO-1 expression from joint homogenates of vehicle-fed, EGCG-fed or EGCG-fed + 1-MT treated CIA mice. **d** Densitometry results for pNrf2, Nrf2 and HO-1 are shown for (**c**) as relative fold change compared to the GAPDH loading control. Results are representative of three independent experiments, with 4 to 5 mice per group. **e** Immunohistochemical staining, original magnification, X200, for pNrf2 in knee sections from vehicle-fed, EGCG-fed or EGCG-fed + 1-MT treated CIA mice (*upper panel*). Cartilage degradation was revealed by safranin-O staining of paw sections from vehicle-fed, EGCG-fed or EGCG-fed + 1-MT treated CIA mice (*lower panel*). Results are representative of three independent experiments

Nuclear factor, erythroid 2-like 2 (Nrf-2) is a transcription factor that plays a major role in cellular defense against oxidative stress by inducing proteins, such as Heme oxygenase-1 (HO-1), that inactivate reactive oxygen species. Increased levels of Nrf-2 result in enhanced antioxidant activity, which then suppresses inflammation in several animal models [34–36]. Although increases in total Nrf2 expression was variable in joint homogenates, there was a consistently dramatic enhancement of phosphorylation of Nrf2 (p-Nrf2), indicating pNrf2 activation, observed in arthritic joint homogenates of EGCG-fed compared with vehicle-fed CIA mice (Fig. 7a). The level of p-Nrf2 was significantly elevated in EGCG-fed CIA mice compared with control vehicle-fed mice as determined by densitometry Fig. 7b. We also examined the expression of HO-1, a downstream target of Nrf2. In EGCG-fed animals, HO-1 protein was significantly increased compared with vehicle-fed CIA mice and by densitometry EGCG treatment increased total HO-1/GAPDH ratios relative to control vehicle-fed mice (Fig. 7a, b).

IDO plays a pivotal role in the inflammatory pathology associated with systemic autoimmunity and disease progression [32]. We next examined whether the function of IDO was in part related to the activation of Nrf2 observed in these mice. Thus, EGCG-fed CIA mice were treated with the IDO inhibitor 1-MT for three weeks and monitored for the development and severity of arthritis. 1-MT treated EGCG-fed mice displayed arthritis that was similar to the vehicle-fed control CIA mice. Immunohistochemical analysis of knee joints demonstrated that p-Nrf2 expression was enhanced in EGCG-fed CIA mice and that this increased expression was blocked by 1-MT (Fig. 7e). Examination of Safranin-O stained paws from CIA mice demonstrated that EGCG-fed mice were protected from cartilage degradation in contrast to vehicle-fed and EGCG-fed mice treated with 1-MT (Fig. 7e). In further support of these results, p-Nrf2, Nrf2 and HO-1 from joint homogenates were examined by western blot (Fig. 7c, d). 1-MT-treated EGCG-fed CIA mice had similar levels of all three molecules as compared to vehicle-fed control CIA mice. EGCG-fed mice expressed increased levels of p-Nrf2 as well as total Nrf2 and HO-1. These results are consistent with an IDO-dependent mechanism of enhanced Nrf2 expression in the inflamed joints of CIA mice.

## Discussion

Rheumatoid arthritis (RA) is a chronic inflammatory disease characterized by T-cell mediated inflammation which contributes to the destruction of cartilage and bone in the joints [14–16, 25]. In animal models, CIA induced by injection of type II collagen in complete Freund’s adjuvant (CFA), shares a number of relevant features with human RA. By evaluating the molecular and cellular effects of green tea polyphenols or EGCG in different autoimmune models, these studies have brought to the forefront the beneficial effects of EGCG in modulating inflammation in animal models of experimental autoimmune encephalomyelitis (EAE) [18], lupus-like and other immune-mediated glomerulonephritis [17, 35], spontaneous non-obese diabetic mice [37] and Sjogren’s syndrome [38]. Our studies indicated that green tea extract or EGCG administration improved symptoms of arthritis, pathological features, and decreased serum CII-specific IgG2a antibody levels. In addition, EGCG treatment markedly reduced inflammation-related cytokine production including IFN-γ, IL-6 and TNF-α whereas it increased the production of IL-10. The present report adds mechanistic insights to previous observations that EGCG can be beneficial in arthritis models [20].

EGCG has been reported to modulate lymphocyte, neutrophil, macrophage and dendritic cell functions [16, 21, 39–43]. In *in vitro* cultures, EGCG altered B and T cell proliferative responses [16, 41, 43, 44]. Using a mixed lymphocyte culture assay it was evident that green tea extract significantly inhibited the proliferation of murine lymphocytes after stimulation with a potent T cell mitogen [45]. Our studies demonstrate that the total number of cells isolated from dLN were significantly lower in EGCG-treated than vehicle-fed CIA mice (*P* < 0.01). EGCG also suppressed the proliferation of autoreactive T cells and to a lesser extent, mitogen-stimulated T cells as measured by ^3^H-thymidine incorporation. These studies parallel the changes observed in the CII antigen-specific T cell division assays using the tracking dye CFSE, which indicated that EGCG suppressed T cell proliferation. In a previous study, it was found that EGCG might inhibit T cell proliferation through modulating the IL-2/IL-2R system [43]. IL-2 can induce IFN-γ production and IL-2 blockade can lead to the inhibition of IFN-γ production [28, 43, 45], and this might underlie some of the observations in the current study. Recently, it was found that EGCG also inhibits B lymphocyte proliferation and induces B lymphocyte apoptosis [44]. Consistent with this, we found that EGCG administration reduced total dLN B cell (CD5^−^B220^+^) frequencies as well as percentages of major splenic B cell subsets including follicular (Fo, CD21^int^CD23^hi^) and marginal zone (MZ, CD21^hi^CD23^lo^) B cells. Taken together, we conclude that EGCG suppresses both T and B cell expansion induced by CII in arthritic mice as EGCG effected both the frequency and absolute numbers of cells.

Previous reports have described the correlation between the decreased function and/or percentage of CD4^+^CD25^+^ Treg cells in patients with RA and clinical disease activity [46]. Moreover, CD4^+^CD25^+^ T cells isolated from arthritic animals were capable of exerting suppressor function in *in vitro* assays [47], while it has been shown that the depletion of CD4^+^CD25^+^ cells could lead to the spontaneous development of autoimmune diseases and increased severity of symptoms in CIA mice [48]. In an EAE model, EGCG also reduced the production of IFN-γ, IL-17, IL-6, IL-1β and increased Treg numbers in lymph nodes and spleen [18]. In agreement with previous findings, the current study showed that EGCG–fed mice exhibited increased percentages of CD4^+^ CD25^+^ Foxp3^+^ Treg cells when compared with vehicle-fed CIA mice. These results are also consistent with the findings of other investigators who have shown that *in vitro* treatment with EGCG modestly enhances Foxp3 and IL-10 mRNA expression in a CD4^+^ T cell line [49].

This study is in agreement with previous reports that IDO expressing innate immune cells can help generate Tregs. For example, human IDO expressing plasmacytoid DC triggered by TLR ligation induced the generation of CD4^+^CD25^+^Foxp3^+^ Tregs from CD4^+^CD25^−^ T cells [50]. Other studies also support this result as CD11c^+^CD11b^+^ DCs isolated from peyer's patches of orally tolerized mice with type II collagen appeared to be necessary for the expansion and differentiation of CD4^+^CD25^+^ T cells, which suppressed CII-specific T-cell proliferation [31]. These studies support our observation that CD11b^+^ DCs isolated from EGCG-fed mice express high levels of IDO and can facilitate the generation of Ag-specific CD4^+^CD25^+^ Foxp3^+^ Tregs. Blocking IDO activity with the specific inhibitor, 1-MT, significantly abrogated the proportion of CD4^+^CD25^+^ Foxp3^+^ T cells induced by CD11b^+^ DCs from EGCG-fed CIA mice. Thus, these data support the hypothesis that EGCG-induced IDO-expressing CD11b^+^ DCs can generate Tregs from CD4^+^CD25^−^ cells in CIA mice.

Previously, antioxidants and antioxidative enzymes have been shown to reduce cartilage damage in animal models of RA, with Nrf2 being a major player [51, 52]. Moreover, Nrf2 deficiency leads to an acceleration of the effector phase of arthritis [52]. It has also been reported that Nrf2 activity inversely correlates with disease in RA patients [53]. Our data demonstrate that EGCG treatment significantly increased pNrf2 activity, and increased expression of HO-1, a Nrf2 target gene. Devesa and colleagues have reported that induction of HO-1, which is protective against joint destruction, can exert partial anti-arthritic effects in CIA [54]. Recently, it has been found that pharmacological up-regulation of HO-1 causes a robust anti-inflammatory response in a model of non-autoimmune arthritis in mice [55], and might prove to be a novel therapeutic target in treatment of chronic inflammatory diseases. Thus, the finding that EGCG enhanced Nrf2 activity resulting in increased levels of HO-1 is of considerable significance.

Of note, activation of Nrf2 in T cells by *tert*-butylhydroquinone (tBHQ), inhibits production of the Th1 cytokine, IFN-γ [56]. Maicas et al. reported that deficiency of Nrf2 resulted in increased migration of pro-inflammatory cells into the joints during the development of arthritis, with significant elevations in TNF-α and IL-6 levels compared with wild type controls [51]. This is also consistent with reports indicating that CD4^+^ T cells from Nrf2 null mice secreted increased amounts of IFN-γ whereas levels of IL-4, IL-5 and IL-13 are decreased [51]. In addition, several reports have demonstrated that a deficiency in Nrf-2 activity results in greater sensitivity to oxidative and inflammatory disorders such as asthma, colitis and sepsis [57–59]. Recently, a chronic granulomatous disease (CGD) patient was found with undetectable IDO metabolic activity, increased Th17 cells as well as impaired transcription factor Nrf2 activity [60]. CGD is an inherited immunodeficiency characterized by a hyper-inflammatory response and an inability to produce reactive oxygen intermediates (ROI), which might lead to impaired counter-regulation by the IDO pathway and insufficient Nrf2 activation [32, 61]. Interestingly, our data revealed that EGCG-fed CIA mice had significantly decreased levels of IFN-γ IL-1β, IL-6 and TNF-α and increased IL-10 levels in joint homogenates and serum as compared to vehicle-fed CIA mice supporting the observation that increased Nrf2 activity correlates with suppression of the inflammatory response.

Collectively, our studies and previous work support a model whereby EGCG-induced Nrf2 activation may skew T cells from a Th1/Th17 phenotype to a Th2 and Treg phenotype and this finding warrants further investigation. The current study is the first to report a relationship between the effects of EGCG treatment and the induction of IDO expression, an activity which can then upregulate antioxidant pNrf-2 activity in mice with arthritis. Further studies are required to determine the clinical relevance of these findings and a systematic testing of potential therapeutic targets in this regulatory cascade.

## Abbreviations

Ag: Antigen
BSA: Bovine serum albumin
CII: Bovine type II collagen
CFA: Complete Freund's adjuvant
CIA: Collagen-induced arthritis
DC: Dendritic cells
dLN: Draining lymph node
DTT: Dithiothreitol
EAE: Experimental autoimmune encephalomyelitis
EGCG: Green Tea (−)-epigallocatechin-3-gallate
EDTA: Ethylenediaminetetraacetic acid
ELISA: Enzyme-linked immunosorbent assay
HBSS: Hank’s balanced salt solution
HO-1: Heme oxygenase-1
HRP: Horseradish peroxidase
IDO: Indoleamine‐2,3‐dioxygenase
1MT: 1-methyl tryptophan
Nrf-2: Nuclear Factor, Erythroid 2-Like 2
PBS: Phosphate buffered saline
PBST: Phosphate buffered saline with Tween 20 buffer
RA: Rheumatoid arthritis
Treg: Regulatory T cells
